# BCG Vaccination Status, Age, and Gender as Risk Factors for Leprosy in Endemic Areas in the Brazilian Amazon

**DOI:** 10.3390/idr12030019

**Published:** 2020-11-27

**Authors:** Luana Nepomuceno Gondim Costa Lima, Jasna Letícia Pinto Paz, Maria do Perpétuo Socorro Corrêa Amador Silvestre, Letícia Siqueira Moura, Ismari Perini Furlaneto, Karla Valéria Batista Lima

**Affiliations:** 1 Bacteriology Section, Instituto Evandro Chagas, Ananindeua 67030-000, Brazil; socorroamador@iec.gov.br (M.d.P.S.C.A.S.); letysiq@outlook.com (L.S.M.); karlalima@iec.gov.br (K.V.B.L.); 2 Ph.D. Program in Parasitic Biology in the Amazon, Universidade do Estado do Pará, Belém 66087-310, Brazil; jasnapaz@yahoo.com.br; 3 Department of Medicine, Centro Universitário do Estado do Pará, Belém 66065-205, Brazil; ismaripf@hotmail.com

**Keywords:** leprosy, mycobacterium leprae, epidemiology, BCG

## Abstract

In 2018, 208,619 new cases of leprosy were reported to the World Health Organization (WHO). Of these, 30,957 occurred in the Americas region and 28,660 (92.6% of the total in the Americas) were reported in Brazil. This study aimed to show the reality of the profile of a population in an endemic leprosy area in northern Brazil in relation to age, gender, and bacillus Calmette–Guérin (BCG) vaccination status through the collection of data in the field with the evaluation of the study individuals, who were recruited by spontaneous demand. A total of 405 individuals participated in the study, with 100 multibacillary, 57 paucibacillary, and 248 healthy contacts. A relationship was observed between the occurrence of the disease, as well as the multibacillary form with the largest age group. The male gender was associated with leprosy per se, with the multibacillary form and was the largest representative of the group that was not vaccinated once. BCG vaccination was effective both in protecting against leprosy per se and in the multibacillary form. These results are limited by sample size, may not be conclusive, and will need further confirmation in a larger cohort.

## 1. Introduction

Leprosy is an infectious, transmissible, and chronic disease. Its etiologic agent is the *Mycobacterium leprae*, a bacillus that mainly affects the peripheral nerves, eyes, and skin. The disease affects people of both gender and any age group; may have a slow and progressive evolution; and, when left untreated, can cause deformities and physical disabilities that are often irreversible [1].

In 2018, 208,619 new cases of the disease were reported to the World Health Organization (WHO). Of these, 30,957 occurred in the Americas region and 28,660 (92.6% of the total in the Americas) were reported in Brazil, in which leprosy persists as a public health problem. Between 2014 and 2018, 140,578 new leprosy cases were diagnosed in Brazil, among which 77,544 cases occurred in males (55.2%). In the same period, male predominance was observed in most age groups. The largest number of cases was identified in individuals between 50 and 59 years old, totaling 26,245. In 2019, Brazil diagnosed 23,612 new leprosy cases, and 78.2% were classified as multibacillary. Of the new cases, 1319 (5.6%) were in children under 15 years old. Given this scenario, Brazil is classified as a country with a high burden for the disease, ranking second in the list of countries with the highest number of cases in the world, behind only India [2,3,4].

In 1991, the Brazilian Ministry of Health officially recommended that home contacts of leprosy patients be revaccinated with BCG (bacillus Calmette–Guérin) to increase the effectiveness of the first dose administered to newborns as a prophylactic vaccine for tuberculosis. The justification for using BCG as a leprosy vaccine rests on the knowledge that *M. leprosy* and *M. bovis* (BCG) share many antigens with a high degree of homology. Contacts without a BCG scar or with just one are vaccinated, and with two scars they are not vaccinated. Currently there are still doubts as to whether BCG protects from leprosy [5,6,7].

Currently, the fight against leprosy is a priority for the Ministry of Health of Brazil, and the epidemiological knowledge of the disease in the region and the vaccination status of individuals with BCG is an important tool to outline action strategies for the early detection of cases in order to prevent physical disabilities and promote a break in the transmission chain. Thus, this study aimed to show the reality of the profile of a population in an endemic leprosy area in northern Brazil in relation to age, gender, and BCG vaccination status through the collection of data in the field with the evaluation of the study individuals, who were recruited by spontaneous demand.

## 2. Methodology

### 2.1. Sampling and Data Collection

The study included individuals from four leprosy endemic municipalities in the State of Pará in northern Brazil: Rondom do Pará, Curianópolis, Goianésia, and Redenção. The participants were divided into two groups of patients and contacts, and recruitment was carried out on spontaneous demand at the municipal health centers.

The group of patients was composed of individuals with a clinical diagnosis of leprosy, undergoing treatment from three months to five months, who at the time of diagnosis were classified according to the Ministry of Health of Brazil into paucibacillary (PB) or multibacillary (MB) [2,8]. The contact group consisted of individuals who lived with leprosy patients and did not have clinical symptoms of leprosy. Thus, 405 individuals participated in the study, with 100 multibacillary, 57 paucibacillary, and 248 healthy contacts.

Data on age, gender, and BCG vaccination were collected using the participants’ identity document and vaccination card.

All the participants provided written informed consent and the study was approved by the municipal health authorities and the Research Ethics Committee of the Evandro Chagas Institute (Ministry of Health) (CAAE 48723115.1.0000.0019).

### 2.2. Statistic

The proportions observed within each studied group were analyzed with the aid of GraphPad Prism version 8.00 (https://www.graphpad.com/scientific-software/prism/), using the G, Chi-square, and Fisher’s Exact tests to verify the association between variables displayed in a 2 × 2 table. The Odds Ratio association measure was used to assess the association between exposure and outcomes of interest. Values of *p* ≤ 0.05 were considered significant.

## 3. Results

Regarding the age group, there was a significantly higher frequency of individuals aged 32 years or older among patients and individuals aged up to 31 years between contacts (*p* < 0.0001). In addition, all age groups from 16 years of age were associated with greater chances of developing leprosy compared to children and adolescents aged 0 to 15 years (Table 1).

Considering only the patients, there was a significantly higher frequency of individuals aged over 46 years among the MB, and, among the PB, there was a predominance of individuals aged between 32 and 46 years (54.0% and 50.9%, respectively, *p* = 0.0210). The chance of presenting as MB or PB in the 0 to 15, 16 to 31, and 32 to 46 age groups was statistically similar, and being over 46 years old increased the chance of presenting the MB form three times (OR = 3.375; 95% CI = 0.718–15.280) (Table 1).

With regard to gender, more women were observed between contacts and more men among patients (66.5% and 51.0%, respectively, *p* = 0.0006; Table 1), and being male was associated with doubling the chance of presenting leprosy (OR = 2.065; 95% CI = 1.369–3.073). Considering the disease classification, there was a higher frequency of male patients among MB (58.0%, *p* = 0.0212), and it was observed that male gender was related to an increase of approximately 2.1 times in the chance of manifesting the MB form of leprosy (OR = 2.197; 95% CI = 0.121–4.245).

With regard to the total doses of BCG vaccine received, there was a significantly higher frequency of contacts among those who received one dose of the vaccine and a higher frequency of patients among those who were never vaccinated (58.5% and 43.9%, respectively, *p* < 0.0001; Table 1). Among MB patients, there was a higher proportion of individuals who had never received BCG (52.0%) when compared to PB (*p* = 0.0183; Table 1).

Individuals who received one or no dose of the vaccine were significantly more likely to develop leprosy when compared to those who received two or more doses, and the group that did not receive any dose of the vaccine had an 8.4-fold increased chance (OR= 8.431, 95% CI= 4.016–17.260; Table 1). Considering the chance of being multibacillary, this was significantly higher among patients who were never immunized with BGC when compared to the chance of this event status among those who took two or more doses (OR = 4.200, 95% CI= 1.180–13.740; Table 1). Considering the gender and vaccination status of the participants, a significant association was found between the absence of immunization and the male sex and between the female sex and the receipt of two or more doses of BCG (*p* < 0.0001; Table 1).

## 4. Discussion

The higher prevalence of individuals over 46 years of age in the patient group and an 8.7-times greater chance of developing the disease in that group (OR = 8.792) corroborates with previous studies which show that it is common in Brazil that leprosy has its highest concentration rates among the older age groups [9]. The long bacillus incubation period, the lack of diagnosis, and/or a late diagnosis can be influencing factors on the high number of older people with the disease [10].

Likewise, the multibacillary manifestation in this study stood out in individuals over 46 years old, in line with the research by Nobre et al. (2017), who sought to identify groups at higher risk for the multibacillary form of leprosy in Brazil, in which they describe that more than half of the Brazilian states have the highest numbers of multibacillaries as age increases. The authors associated this relationship of multibacillary activity with older age agrees with the hypothesis that leprosy transmission is decreasing and that these cases reflect a transmission that occurred a while ago and not recently, because *M. leprae* has a long incubation period [11].

Silva et al. (2018) analyzed the new cases reported from a city in the state of Maranhão (northeast of Brazil) in the period between 2003 and 2015 and observed that most individuals aged between 50 and 59 years old (59.2%) and over 60 years (72.3%) were within the MB classification. The authors proposed as a possible explanation for the high number of sick people at older ages—in addition to the incubation period—the delay in diagnosing these patients, a factor that keeps them as active sources of infection transmission for long periods of time. This common delay in the diagnosis of leprosy in developing countries was observed in an epidemiological and clinical study with elderly people over 60 years of age in southern Brazil, in which almost 40% of the patients were at grade II disability, which is a classic late-diagnosis feature [12,13].

In the population of the present study, the contact group was predominantly female, while in the patient group and the multibacillary group there was a male predominance (*p* < 0.05). Likewise, the chances of developing the disease were two times greater for males (OR = 2.065), and the chances of developing the multibacillary form were two times greater for this gender (OR = 2.197). The relationship found by this research between the male sex and the occurrence of leprosy and, specifically, the development of the MB form of the disease has also been observed by research conducted in northeastern Brazil [9]. This relationship raises the social issues surrounding men’s health, such as the image of virility and the incompatibility of the opening hours of health units with the free time of these individuals (after their work routine), but it may also be influenced by the physiological differences between men and women, because testosterone has immunosuppressive activity in both humoral and cellular responses, while estrogen stimulates the production of TNFα, an important cytokine in the Th1 cellular response. Thus, men and women can be equally exposed to the bacillus, with a higher proportion of men among the cases due to men having some susceptibility to developing the disease after being infected [14,15,16,17].

Another explanation may be due to a greater exposure of men to the environment compared to women, since men are more engaged in professions that come into contact with soil, water, and animals. A study carried out in Brazil demonstrated that males had increased chances of having *M. leprae* DNA in the nose by 6.2 times, which may reflect a greater probability of sex in acquiring the disease [18]. In a cohort study by Bakker et al. (2006), it was shown that males are twice as likely to develop leprosy compared to women [19].

Evidence suggests that BCG may have beneficial effects in the treatment of câncer (non-invasive bladder), autoimmune diseases (Type 1 diabetes, multiple sclerosis), and Alzheimer’s disease. This probably occurs through heterologous immunity such as protection against undirected pathogens in addition to the targeted pathogen, the activation of heterologous lymphocytes, and innate immune memory. BCG heterologous immunity can be used to improve outcomes in vulnerable populations, particularly the very young and the elderly [20,21,22,23].

Thus, despite the non-specificity of the bacillus Calmette–Guérin (BCG) vaccine for *M. leprae*, it is approximately 50% effective in protecting against leprosy [5]. Brazil, together with Colombia, Peru, and Australia, makes up the list of a few countries that use the application of a second dose of BCG vaccine for the contacts of new cases of leprosy as a national prophylactic measure. Although not recommended by the World Health Organization (WHO), the application of this vaccination specifically for leprosy prophylaxis is recognized by the WHO for its significant contribution to the decline in the incidence of the disease [7,24]. The BCG vaccine induces the activation of T-cell clones that recognize specific M. leprae epitopes, providing a protective effect against disease progression, including leading to negative PGL-I serological tests that were previously positive [6,25].

In the present study, 83.33% of the healthy household contacts of patients were vaccinated at least once. As for the patients, almost half did not receive any dose of the vaccine (Table 1). The group of individuals who did not take any dose of the vaccine was 8.4 times more likely to develop leprosy (OR = 8.431). Studies point out that the second BCG vaccination increases protection against the disease, reaching a 95% decrease risk in the relative risk with the application of the second dose [24,26]. Among the patients participating in the present study, only 7.1% had received two doses of the vaccine.

In addition, observing the general vaccination situation of the individuals in the study, there is a divergence by gender. The greater number of female individuals vaccinated in relation to the male sex corroborates the idea that, in general, women are more willing to take care of their health than men [15]. A similar result was observed in a survey conducted in the southeast of Brazil, in which the percentage of men who did not receive doses of the vaccine was higher than the percentage of women [27].

Thus, BCG vaccination seems to be an indispensable component of any program that aims to control or eradicate leprosy, because, despite important advances in the study of the molecular biology of *M. leprae*, specific vaccines against leprosy are still at an early stage initial development and evaluation. Although both BCG vaccination and treatment with the index case reduce the risk of contacts contracting leprosy, the changes in the immune response induced by these two measures, which could explain the resulting protective effect, still need to be investigated in detail. Thus, in view of the complex current difficulties encountered in eradicating this disease, we continue to need urgent measures to reveal the hidden side of the leprosy “epidemiological iceberg” in order to reduce its morbidity and the physical disabilities resulting from this disease.

## 5. Ethics Approval and Consent to Participate

All the participants provided written informed consent, and the study was approved by the municipal health authorities and the Research Ethics Committee of the Instituto evandro chagas (Ministry of Health of Brazil) with the committee reference number 48723115.1.0000.0019.

## 6. Conclusions

A relationship was observed between the occurrence of the disease and the multibacillary form and the largest age group. The male gender was associated with leprosy per se and with the multibacillary form and was the largest representative of the group that was not vaccinated once. BCG vaccination was effective both in protecting against leprosy per se and the multibacillary form. These results are limited by sample size, may not be conclusive, and will need further confirmation in a larger cohort.

## Figures and Tables

**Table 1:** Characterization of groups regarding age, gender, and doses of BCG vaccine.

	Patient	Contacts	OR (IC 95%) *	*p*-Value	MB	PB	OR (IC 95%) *	*p*-Value **
**Age group**								
**0–15**	6 (3.8%)	52 (21.0%)	1 (-)	<0.0001 ^†^	3 (3.0%)	3 (5.3%)	1 (-)	0.0210 ^†^
**16–31**	19 (12.1%)	57 (23.0%)	2.889 (1.070–7.631 ^†^)		10 (10.0%)	9 (15.8%)	1.111 (0.214–5.726)	
**32–46**	62 (39.5%)	70 (28.2%)	7.676 (3.244–17.980 ^†^)		33 (33.0%)	29 (50.9%)	1.138 (0.249–5.164)	
**>46**	70 (44.6%)	69 (27.8%)	8.792 (3.514–20.480 ^†^)		54 (54.0%)	16 (28.0%)	3.375 (0.718–15.280)	
**Total**	157 (100%)	248 (100%)			100 (100%)	57 (100%)		
**Gender**								
**Female**	77 (49.0%)	165 (66.5%)	1 (-)	0.0006 ^†^	42 (42.0%)	35 (61.4%)	1 (-)	0.0212 ^†^
**Male**	80 (51.0%)	83 (33.5%)	2.065 (1.369–3.073 ^†^)		58 (58.0%)	22 (38.6%)	2.197 (1.121–4.245 ^†^)	
**Total**	157	248			100	57		
**BCG**								
**0**	68 (43.9%)	41 (16.7%)	8.431 (4.016–17.260 ^†^)	<0.0001 ^†^	51 (52.0%)	17 (29.8%)	4.200 (1.180–13.740 ^†^)	0.0183 ^†^
**1**	75 (48.4%)	144 (58.5%)	2.648 (1.345–5.052 ^†^)		42 (42.9%)	33 (57.9%)	1.782 (0.538–5.519)	
**≥2**	12 (7.7%)	61 (24.8%)	1 (-)		5 (5.1%)	7 (12.3%)	1 (-)	
**Total**	155	246			98	57		

MB: multibacillary. PB: paucibacillary. BCG: dose of bacillus Calmette–Guérin vaccine. * Odds Ratio, with a 95% confidence interval. ** Chi-square or G-test of independence (Chi-square residue analysis), as needed. ^†^ Statistically significant.
